# Nutritional support in bone infections: mechanisms, strategies, and clinical practice

**DOI:** 10.3389/fnut.2026.1972375

**Published:** 2026-10-05

**Authors:** Xiaoqin Li, Can Zhang, Weizhu Zhang, Yun Liang

**Affiliations:** Clinical Medical Center, Affiliated Hospital of North Sichuan Medical College, Nanchong, Sichuan, China

**Keywords:** bone infection, immunonutrition, nutritional support, osteomyelitis, perioperative nutrition, periprosthetic joint infection

## Abstract

**Objective:**

Bone infections, including osteomyelitis and periprosthetic joint infection (PJI), are characterized by prolonged treatment, high recurrence, and functional impairment. Persistent inflammation induces hypercatabolism, negative nitrogen balance, and micronutrient depletion, impairing immune defense and bone repair. Systematic nutritional assessment remains insufficient. This review elucidates metabolic mechanisms under infectious stress, evaluates macronutrients, micronutrients, and immunomodulatory nutrients in infection control and bone regeneration, and proposes a clinical nutritional management framework.

**Methods:**

A structured literature search was conducted in PubMed, Web of Science, and CNKI from January 2020 to April 2026 to inform this narrative review. Studies on metabolic profiles, nutritional interventions, or nutritional status–outcome associations in osteomyelitis or PJI were included. Data were synthesized across five dimensions: molecular mechanisms, macronutrient therapy, micronutrient functions, immunonutrition, and implementation pathways, with evidence graded by OCEBM levels.

**Results:**

TNF-α and IL-6 drive muscle degradation via ubiquitin-proteasome activation and mTORC1 suppression, causing negative nitrogen balance; zinc, selenium, and 25-hydroxyvitamin D are depleted. Nutritional risk markers (albumin ≤ 3.5 g/dl and/or lymphocytes ≤ 1.5 × 10^3^/μl) are associated with 2-−4-fold higher risks of postoperative infection, wound dehiscence, and sepsis, and increased 2-year osteomyelitis risk (RR = 2.55). Protein (1.5–2.0 g/kg/d) and energy (25–35 kcal/kg/d) correct nitrogen balance and support immunity and bone repair. Vitamin D induces cathelicidin via VDR, exerting mineralization and anti-infection effects; zinc/selenium alleviate oxidative damage; omega-3 PUFAs provide anti-inflammatory effects. Probiotics show gut-bone axis potential with limited evidence. High-quality RCTs for bone infections are lacking; most recommendations derive from observational studies (evidence levels C–D).

**Conclusions:**

Nutritional support should be elevated to core status. We recommend routine albumin, prealbumin, and lymphocyte screening, and a “screening → assessment → stratified intervention → dynamic monitoring” pathway. Enteral nutrition is preferred perioperatively, with 1.5–2.0 g/kg/d protein and 25–35 kcal/kg/d energy, plus individualized correction of vitamin D, zinc, and selenium deficiencies. Multicenter RCTs are needed to validate immunonutrient efficacy and advance precision nutrition via metabolomics and gut-bone axis mechanisms.

## 1 Introduction

Bone infections encompass a heterogeneous group of conditions, including chronic osteomyelitis, periprosthetic joint infection (PJI), diabetic foot osteomyelitis, fracture-related infection, and pressure ulcer-related pelvic osteomyelitis. Among these, chronic osteomyelitis and PJI represent some of the most challenging and refractory diseases in orthopedic practice, characterized by protracted treatment courses, persistently high recurrence rates, and the potential for permanent functional impairment. Traditional therapeutic strategies have primarily focused on surgical debridement and prolonged antibiotic administration. However, accumulating evidence indicates that the patient's nutritional status is a critical independent determinant of treatment outcomes. The infectious state triggers systemic stress and persistent inflammation, upregulating resting energy expenditure, accelerating skeletal muscle protein catabolism, and substantially depleting micronutrients including zinc, selenium, and 25-hydroxyvitamin D. This metabolic derangement not only impairs the host's immune capacity to clear pathogens but also directly suppresses osteoblast activity, thereby blocking bone defect repair. Consequently, nutritional support should not be relegated to an adjunctive role but rather integrated as a core component throughout the entire treatment pathway for bone infections.

Clinical research has further substantiated the close relationship between nutritional status and prognosis in bone infections. A study of pressure ulcer-related pelvic osteomyelitis found that treatment failure (requiring repeat surgery or intravenous antimicrobial therapy) occurred in 71% of cases, with an ultimate ulcer healing rate of only 36% and a one-year mortality rate of 23% (1). Multivariable analysis identified lack of formal nursing support upon discharge, post-traumatic neurological deficits, and elevated admission C-reactive protein (>184 mg/L) as significantly associated with treatment failure, while age (>59.5 years), failure to attempt soft tissue coverage, hemoglobin (< 111 g/L), and albumin (< 25 g/L) were independent risk factors for ulcer non-healing. It is important to recognize that serum albumin is influenced by multiple factors beyond nutritional intake, including systemic inflammation, capillary leakage, hepatic acute-phase reprogramming (as IL-6 suppresses albumin synthesis during infection), hydration status, and disease severity. Therefore, hypoalbuminemia should be interpreted as a prognostic marker reflecting both inflammatory burden and nutritional reserve, rather than as a direct measure of isolated protein deficiency—a distinction particularly important in bone infections where inflammation can be substantial. It should be noted that these findings are based on observational data; although they reveal significant correlations between nutritional parameters and adverse outcomes, they have not directly validated the therapeutic effect of nutritional interventions on prognosis. Nevertheless, these observations support the incorporation of laboratory nutritional markers into perioperative risk stratification for patients with bone infections. Another retrospective cohort study of patients undergoing humeral shaft fracture surgery utilized laboratory-defined nutritional risk markers (serum albumin ≤ 3.5 g/dl and/or lymphocyte count ≤ 1.5 × 10^3^/μl) for stratified analysis. Patients with positive nutritional risk markers had significantly higher risks of acute respiratory failure, venous thromboembolism, postoperative infection, wound dehiscence, sepsis, and acute kidney injury within 90 days post-surgery (all *p* < 0.001) (2). During the 2-year follow-up period, this group also demonstrated significantly increased risks of infection, implant removal, osteomyelitis, and bone grafting. These findings support the potential utility of laboratory nutritional markers for risk stratification in orthopedic trauma patients, and suggest that similar approaches warrant investigation in patients with bone infections.

The importance of nutritional support is particularly pronounced in complex cases such as diabetic foot ulcer complicated by osteomyelitis. Biomechanical alterations due to structural foot deformities predispose patients to plantar ulcers, and ulcer recurrence frequently leads to osteomyelitis. Once infection spreads, treatment becomes extremely complex and difficult, primarily due to poor circulation and inadequate local tissue nutrition resulting in suboptimal antibiotic concentrations (3). Furthermore, the peri-ulcerative tissue is scant and thin, and elevated plantar pressure impedes granulation tissue formation; concurrent hyperkeratosis of the peri-wound skin prevents epidermal regeneration, and poor vascularity often renders skin grafting unsuccessful. Thus, managing such complex wounds and preventing recurrence is technically challenging. A successful case report demonstrated wound healing without recurrence through combination therapy including surgical bone resection, vancomycin-impregnated cement implantation, negative pressure wound therapy, autologous platelet-rich gel, and customized offloading footwear for preventive foot care (3). The success of this comprehensive management strategy not only relied on surgical and antibiotic therapy but also highlighted the central value of improving the local nutritional microenvironment and promoting tissue regeneration in bone infection treatment.

Notably, the challenge of nutritional support in bone infections extends beyond adults to include pediatric populations with chronic wounds, including osteomyelitis. Although recent studies have noted an increasing number of pediatric chronic wound cases, the epidemiology and management strategies in this population remain understudied. A systematic review reported that osteomyelitis accounts for approximately 13% of pediatric chronic wound cases, with management often requiring multidisciplinary collaboration involving pediatric surgery, dermatology, and allied health professionals (4). The review emphasized that most epidemiological studies consider demographic factors, nutritional status, and comorbidities (such as diabetes, obesity, pilonidal disease, and myelomeningocele). This indicates that nutritional status is a critical factor in assessing bone infection risk and formulating treatment strategies in both adults and children. Bone infections comprise a heterogeneous group of diseases that vary significantly in terms of patient demographics, pathogen spectrum, inflammatory burden, soft tissue and vascular status, the presence of implant materials, duration of antibiotic therapy, surgical management, and metabolic complications. The nutritional principles discussed in this review are broadly applicable across these subtypes, but the strength of evidence and specific implementation may vary. For example, the evidence for nutritional interventions in diabetic foot osteomyelitis is largely extrapolated from diabetic wound care literature, whereas evidence in PJI is derived primarily from general arthroplasty populations. Where subtype-specific evidence is available, distinctions are noted; otherwise, the recommendations should be interpreted as general frameworks requiring individualization based on the specific clinical context. Future research should further define the specific nutritional requirements of patients with bone infections across different age groups and explore more effective nutritional intervention strategies to improve outcomes in this complex disease.

This review is presented as a narrative review rather than a formal systematic review. Given the heterogeneity of the available evidence—which spans basic mechanistic studies, animal models, observational clinical data, and extrapolated guidelines—a narrative synthesis approach was chosen to integrate these diverse sources into a clinically meaningful framework. A structured literature search was conducted to inform the review (see Literature Search Strategy below), but the primary aim was to provide a comprehensive overview and critical interpretation of the field rather than to answer a narrowly defined PICO question through quantitative synthesis.

## 2 Literature search strategy

This narrative review was informed by a structured literature search conducted in PubMed, Web of Science, and China National Knowledge Infrastructure (CNKI) from January 2020 to April 2026. The search strategy combined terms related to bone infection (“osteomyelitis,” “periprosthetic joint infection,” “diabetic foot osteomyelitis,” “bone infection”) with nutritional terms (“nutritional support,” “nutrition therapy,” “micronutrients,” “vitamin D,” “zinc,” “selenium,” “omega-3,” “glutamine,” “arginine,” “probiotics,” “immunonutrition”). Boolean operators (AND/OR) were used to combine search terms. Additional relevant articles were identified through manual screening of reference lists of included studies and through citation tracking of key reviews. Given the broad and heterogeneous nature of the available evidence—which includes mechanistic studies, animal models, observational clinical data, and extrapolated guidelines—this review is presented as a narrative synthesis with the aim of integrating diverse evidence sources into a clinically meaningful framework. The evidence was organized thematically and interpreted with attention to the strength and directness of each cited study relative to bone infection populations.

### 2.1 Inclusion and exclusion criteria

Studies were considered for inclusion if they met the following criteria: (1) the study population comprised patients with osteomyelitis, periprosthetic joint infection, diabetic foot osteomyelitis, or other bone infections; (2) the study content addressed nutritional metabolism, nutritional assessment, nutritional intervention, or the association between nutritional status and clinical outcomes; (3) the study type was original research (including randomized controlled trials, cohort studies, and case-control studies), systematic review/meta-analysis, or high-quality narrative review; and (4) the article was published in English or Chinese. Studies were excluded if they were (1) single case reports (unless of exceptional teaching value), (2) conference abstracts, comments, or letters to the editor, or (3) duplicate publications or studies with incomplete data.

### 2.2 Evidence grading

Evidence was interpreted using the Oxford center for evidence-based medicine (OCEBM) 2011 Levels of Evidence framework as a reference. Each cited study was considered according to its study design and directness to bone infection populations. For the purposes of this review, evidence was consolidated into a simplified three-tier grading for clinical applicability:

Grade B: Consistent OCEBM Level 1 or 2 studies (randomized controlled trials or systematic reviews of RCTs). Note: In this review, Grade B evidence for glutamine is derived from RCTs in critically ill populations and does not reflect bone-infection-specific RCT evidence.

Grade C: OCEBM Level 3 or 4 studies (cohort studies, case-control studies, or mechanistic studies), or extrapolations from higher-level studies in different populations.

Grade D: OCEBM Level 5 evidence (expert opinion, basic physiological principles, or case reports), or insufficient/controversial evidence.

Evidence interpretation was performed by the first author and reviewed by the corresponding author.

## 3 Metabolic derangements and nutritional requirements in bone infection

### 3.1 Hypermetabolism and hypercatabolism

Bone infections (such as osteomyelitis and implant-associated infections) trigger a robust stress response, inducing systemic metabolic derangements characterized by hypermetabolism and hypercatabolism. The infection itself and the consequent systemic inflammatory response significantly upregulate resting energy expenditure and accelerate protein catabolism (2). At the molecular level, this state is primarily driven by pro-inflammatory cytokines—particularly tumor necrosis factor-alpha (TNF-α) and interleukin-6 (IL-6). TNF-α and IL-6 bind to their respective receptors on skeletal muscle cell membranes, activating the nuclear factor-kappa B (NF-κB) signaling pathway, which in turn upregulates muscle-specific E3 ubiquitin ligases (such as MuRF-1 and Atrogin-1), initiating the ubiquitin-proteasome pathway and leading to accelerated degradation of myofibrillar structural proteins (5). Concurrently, these inflammatory signals suppress the insulin-like growth factor-1 (IGF-1)/PI3K/Akt signaling axis, thereby attenuating mTORC1-mediated protein synthesis, resulting in a degradation rate that far exceeds synthesis. If this hypermetabolic state persists, it leads to rapid loss of lean body mass, decreased respiratory muscle strength, generalized immune impairment, and directly delays bone defect healing (see Figure 1, immune response pathway module). Cytokine-driven excessive muscle breakdown constitutes the precursor pathological basis for negative nitrogen balance, subsequently triggering systemic protein metabolic reprogramming.

**Figure 1:**
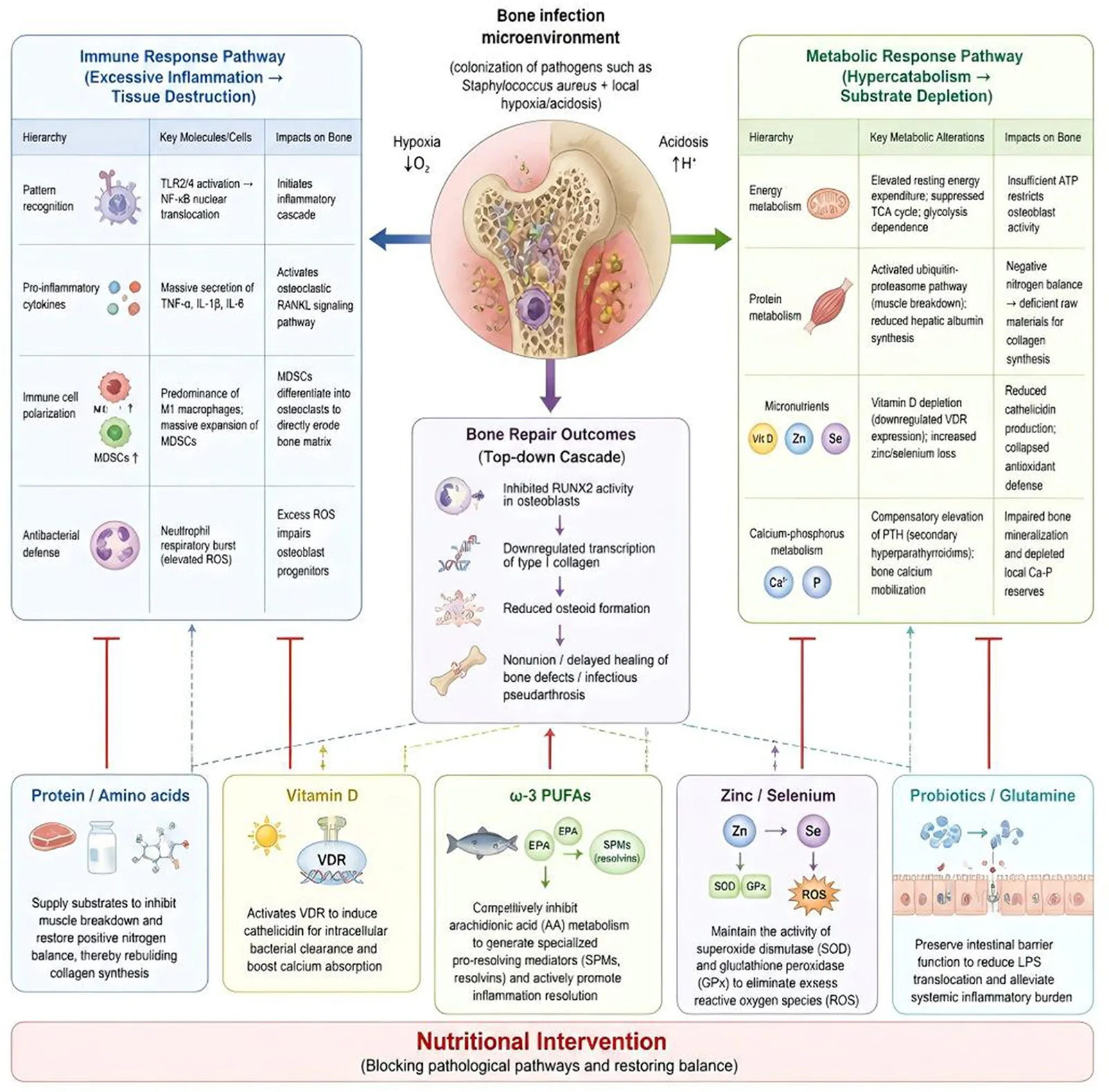
Crosstalk among immune response, metabolic disturbance and impaired bone regeneration during bone infection and the blocking effect of nutritional support. Schematic diagram showing the interactive network of immunity, metabolism and bone repair in bone infection. The bone infection microenvironment, characterized by pathogen colonization, local hypoxia and acidosis, acts as the core node, triggering three pathological cascades: excessive inflammatory immune response, hypercatabolic metabolic disorder, and ultimately suppressed bone repair. Excess pro-inflammatory mediators, reactive oxygen species and nutrient depletion synergistically inhibit osteogenic activity, activate osteoclasts, and lead to bone nonunion, delayed bone healing and infectious pseudarthrosis. Nutritional intervention serves as a comprehensive regulatory hub at the bottom of the network; multiple nutrients exert inhibitory effects on pathological pathways (marked with upward red arrows) by restoring nitrogen balance, enhancing antioxidant capacity, promoting inflammation resolution, strengthening intestinal barrier function and facilitating bone mineralization. TLR, toll-like receptor; NF-κB, nuclear factor κB; TNF-α, tumor necrosis factor-α; IL, interleukin; RANKL, receptor activator of nuclear factor κB ligand; MDSCs, myeloid-derived suppressor cells; ROS, reactive oxygen species; TCA, tricarboxylic acid; VDR, vitamin D receptor; PTH, parathyroid hormone; RUNX2, runt-related transcription factor 2; PUFA, polyunsaturated fatty acid; AA, arachidonic acid; SPMs, specialized pro-resolving mediators; SOD, superoxide dismutase; GPx, glutathione peroxidase; LPS, lipopolysaccharide.

### 3.2 Molecular mechanisms of protein metabolic derangement and negative nitrogen balance

Against the backdrop of hypercatabolism in bone infections, protein metabolic imbalance is particularly prominent, characterized by a systemic protein degradation rate that consistently overwhelms the synthesis rate, with the net effect being progressive negative nitrogen balance. The molecular regulation of this process involves two interrelated components:

#### 3.2.1 First, accelerated degradation of skeletal muscle proteins

In addition to activating the ubiquitin-proteasome pathway, circulating TNF-α and IL-6 enhance intracellular autophagic-lysosomal activity within muscle cells, further accelerating the breakdown of myofibrillar proteins (such as myosin light chain) and releasing large quantities of amino acids into the circulation to meet the urgent substrate demands of hepatic acute-phase protein synthesis and immune cell proliferation (5).

#### 3.2.2 Second, “acute-phase reprogramming” of hepatic protein synthesis

Driven by IL-6 and other signals, the hepatic protein synthesis profile undergoes selective switching—albumin transcription and synthesis are significantly suppressed, while the synthesis of acute-phase reactants such as C-reactive protein (CRP) and serum amyloid A is preferentially upregulated. This metabolic strategy of “sacrificing albumin for acute-phase proteins,” although beneficial for short-term host defense, results in persistently decreased serum albumin levels and exacerbates net nitrogen loss. Furthermore, TNF-α within the bone microenvironment acts directly on osteoprogenitor cells, suppressing mTORC1 signaling and reducing the translational efficiency of type I collagen, thereby further limiting the substrate supply for bone matrix synthesis at the local level (see Figure 1, metabolic response pathway module).

In summary, negative nitrogen balance in bone infections is not simply “inadequate intake” but rather a systemic metabolic disorder driven synergistically by “peripheral muscle breakdown for energy and substrate supply” and “hepatic/local bone synthetic reprogramming,” providing a direct pathophysiological rationale for subsequent adequate protein intervention. Persistent inflammation and protein depletion concurrently deplete micronutrient reserves including copper, zinc, and vitamin D, giving rise to multiple metabolic imbalances.

### 3.3 Micronutrient and vitamin imbalances

During bone infections, the metabolism of multiple trace elements and vitamins is disrupted, and these nutritional imbalances directly affect bone metabolism and immune function. Copper serves as a cofactor for multiple key enzymes involved in energy metabolism, antioxidant defense, and connective tissue synthesis; its homeostatic dysregulation is closely associated with the development and progression of bone infections. Copper overload can induce mitochondrial cuproptosis, affecting osteoblast and osteoclast function, while copper deficiency may impair immune cell function (6). Vitamin D deficiency is particularly common in patients with bone infections; it not only predisposes to metabolic bone disease (such as osteomalacia) but is also associated with secondary hyperparathyroidism (SHPT), leading to increased bone resorption and decreased bone mineral density (7). Additionally, chronic infections such as hepatitis C virus (HCV) infection can lead to vitamin K deficiency, manifested as elevated serum undercarboxylated osteocalcin (ucOC) levels, impairing bone formation (8). In the treatment of bone infections, assessing and correcting deficiencies of these micronutrients (such as copper, zinc, and selenium) and vitamins (such as D, K, and C) is critical for optimizing bone healing, regulating immune responses, and mitigating oxidative stress. Nutrient imbalance, systemic inflammation, and bone cell damage form a vicious cycle, and their interaction can be explained through the “gut-bone axis” mechanism in osteoimmunology.

### 3.4 Immune-metabolic interactions

The core pathophysiological mechanism of bone infections is the complex interaction between the immune system and bone metabolism—termed “osteoimmunology.” The inflammatory response triggered by infection not only directly destroys bone tissue but also profoundly affects bone metabolism through immune cells and the cytokine network. For example, myeloid-derived suppressor cells (MDSCs) expand substantially during chronic inflammation and infection; they can both suppress excessive immune responses through secretion of anti-inflammatory cytokines and, under specific conditions, differentiate into osteoclasts that directly cause bone erosion (see Figure 1, bone infection microenvironment module) (9). Furthermore, the gut microbiota, particularly Lactobacillus species, play an important role in bone remodeling through modulation of immune responses. They transmit immune signals via the “gut-bone axis,” suppressing pro-inflammatory responses and potentially mitigating bone infection-related bone loss (10). The infection itself, such as SARS-CoV-2, can infect bone marrow-derived macrophages through neuropilin-1 (NRP1), inhibiting their differentiation into osteoclasts and thereby disrupting normal bone metabolic balance (11). Therefore, the treatment of bone infections cannot focus solely on pathogen eradication but must also consider modulation of the immune-metabolic network through nutritional support (such as supplementation with specific amino acids and prebiotics) to create an internal environment conducive to bone regeneration and infection control.

## 4 Roles of macronutrients in bone infection treatment

### 4.1 Protein and amino acids: promoting bone repair and immunity

Protein is fundamental to bone tissue repair and immune defense, playing a central role in bone infection treatment. Under bone infection conditions, the body is in a hypercatabolic state with accelerated protein breakdown and suppressed synthesis, leading to negative nitrogen balance. This state not only directly impairs collagen synthesis in the bone matrix, compromising bone repair capacity, but also impairs the proliferation and function of immune cells (such as lymphocytes and phagocytes), thereby reducing the body's ability to clear pathogenic microorganisms. Clinical studies have confirmed that nutritional risk markers (such as hypoalbuminemia) are closely associated with adverse outcomes after orthopedic surgery. For example, a retrospective cohort study of patients undergoing humeral shaft fracture surgery found that those with preoperative positivity for laboratory-defined nutritional risk markers (serum albumin ≤ 3.5 g/dl) had significantly higher rates of infection, wound dehiscence, and sepsis within 90 days post-surgery, as well as significantly increased risks of osteomyelitis and implant removal within 2 years (2). Another study of pressure ulcer-related pelvic osteomyelitis also identified low albumin levels (< 25 g/L) as an independent risk factor for ulcer non-healing (1). These findings indicate that adequate protein intake is essential for maintaining serum albumin levels, supporting immune function, and promoting bone healing. Therefore, in the treatment of bone infections, ensuring adequate protein supply may help meet the increased protein requirements associated with hypercatabolism and mitigate negative nitrogen balance. These targets (1.5–2.0 g/kg/d), which are extrapolated from general critical care and surgical nutrition guidelines, should be considered as initial goals rather than fixed protocols, and should be individualized based on the patient's metabolic status, renal function, and tolerance.

### 4.2 Carbohydrates and fats: optimization of energy substrates

As a severe stress state, bone infection significantly alters the body's energy metabolism pattern. The local infectious microenvironment, particularly in bone tissue, often exhibits hypoxia and metabolic disturbances. Proteomic analysis has shown that in bone tissue from *Staphylococcus aureus*-induced periprosthetic joint infection (PJI), the expression of tricarboxylic acid (TCA) cycle-related enzymes is significantly inhibited, suggesting reprogramming of local energy metabolism pathways (12). Although TCA cycle inhibition is often accompanied by enhanced glycolytic dependence, direct evidence supporting the existence of a “Warburg effect” in the bone infection microenvironment remains insufficient, and this hypothesis requires validation through direct methods such as isotope tracing or metabolic flux analysis. Nevertheless, optimizing energy substrate supply is critical for supporting immune cell function and bone repair processes. Carbohydrates are the primary source of rapid energy; adequate glucose supply can meet the glycolytic demands of immune cells (such as neutrophils and macrophages) during phagocytosis and bactericidal activity. However, excessive carbohydrate intake may lead to hyperglycemia, which paradoxically impairs immune function and increases infection risk. Fats, particularly medium-chain triglycerides (MCT) and omega-3 polyunsaturated fatty acids, not only provide high-density energy but also modulate inflammatory responses. Omega-3 fatty acids promote the production of pro-resolving mediators such as resolvins and protectins, helping to control excessive inflammation and facilitate infection resolution and tissue repair. Therefore, in nutritional support for bone infections, the carbohydrate-to-fat ratio should be rationally adjusted to meet the acute-phase glucose demands of immune cells while utilizing fats for energy provision and inflammation modulation, avoiding the negative consequences of excessive single energy substrate intake.

### 4.3 Special amino acids: immunomodulatory effects of glutamine and arginine

In the metabolic derangements of bone infections, the demand for certain “conditionally essential amino acids” is significantly increased, with glutamine and arginine being particularly notable for their immunomodulatory effects. Glutamine is a critical fuel for immune cells (such as lymphocytes and macrophages) and a precursor for nucleotide synthesis, essential for maintaining intestinal barrier function and antioxidant defense. Under infectious and stress conditions, plasma and tissue glutamine levels decline precipitously, and glutamine supplementation helps enhance immune cell function and reduce infectious complications. Arginine serves as a substrate for nitric oxide (NO) and polyamine synthesis. NO is a key effector molecule for macrophage-mediated pathogen killing, while polyamines participate in cell proliferation and tissue repair. However, arginine metabolism has a dual nature: in host cells, inducible nitric oxide synthase (iNOS) utilizes arginine to produce NO for pathogen killing; whereas in certain pathogens (such as *Leishmania*), their own arginase utilizes arginine to produce polyamines, promoting their own replication (13). This indicates that the metabolic direction of arginine is critical in the host-pathogen interplay. Furthermore, in a murine sepsis model, the amino acid metabolic profile of bone marrow-derived MDSCs showed significant alterations, with elevated levels of leucine, threonine, and glycine, and enrichment in alanine metabolism, glutathione metabolism, and arginine-proline metabolism pathways—changes that may enhance the immunosuppressive function of MDSCs and increase the risk of secondary infection (14). Therefore, targeted supplementation with glutamine and arginine in bone infection treatment may help correct immune imbalance and enhance host defense, but the complexity of arginine metabolism must be carefully considered to avoid providing “nourishment” to pathogens.

## 5 Micronutrients and bone infections

### 5.1 Vitamin D: dual roles in bone metabolism and immune modulation

Vitamin D plays dual roles in bone metabolism and immune modulation in bone infections, with its functions extending beyond traditional calcium-phosphorus homeostasis regulation. In bone metabolism, vitamin D promotes intestinal calcium absorption and maintains serum calcium and phosphorus levels, providing the foundational substrates for bone mineralization; its deficiency directly leads to impaired bone mineralization, increasing fracture and nonunion risk (15). In immune modulation, vitamin D binds to the vitamin D receptor (VDR), inducing antimicrobial peptide (such as cathelicidin) production and enhancing macrophage activity, thereby directly participating in anti-infection immunity (15). Additionally, the vitamin D/VDR signaling pathway exerts anti-infection effects through induction of autophagy, which is crucial for clearing intracellular pathogens (16). Clinical studies have shown that vitamin D deficiency (below 30 ng/ml) occurs in 43%−90% of orthopedic trauma patients and is significantly associated with surgical site infection, periprosthetic joint infection, and delayed fracture healing (15). Vitamin D deficiency is also prevalent in immunocompromised populations such as HIV-infected individuals, correlating with disease progression and increased infection risk (17). Therefore, for patients with bone infections, correcting vitamin D deficiency is not only fundamental to maintaining skeletal health but also an important strategy for enhancing host immune defense and improving infection outcomes.

### 5.2 Zinc and selenium: antioxidant and anti-infection effects

Zinc and selenium, as critical trace elements, exert important effects in bone infections through antioxidant and direct anti-infection mechanisms. Zinc is a cofactor for multiple antioxidant enzymes (such as superoxide dismutase), capable of scavenging excessive reactive oxygen species generated during infection and mitigating oxidative damage to bone tissue. Simultaneously, zinc is essential for normal immune system development and function; its deficiency leads to impaired T lymphocyte function and reduced antibody production, increasing host susceptibility to infection. Selenium, as a core component of selenoproteins (such as glutathione peroxidase), participates in antioxidant defense and protects cell membranes from oxidative damage. In the field of bone repair, the synergistic effects of zinc and selenium have been applied in biomaterial design. For example, 3D-printed selenium/strontium/zinc-doped hydroxyapatite composite scaffolds have demonstrated not only significant anti-tumor effects but also potent antimicrobial potential, effectively inhibiting postoperative infection and promoting bone defect repair (18). Furthermore, the antimicrobial mechanism of selenium partly derives from its ability to induce oxidative stress in bacteria, while zinc exerts bacteriostatic effects through disruption of bacterial cell membrane integrity. Therefore, ensuring adequate zinc and selenium levels in patients with bone infections has important clinical significance for infection control and bone healing promotion. The optimal dosing regimen for vitamin D repletion in bone infection patients has not been established. The doses listed in Table 1 are extrapolated from general clinical practice and should be individualized based on baseline 25-hydroxyvitamin D levels, renal function, and serum calcium monitoring. A loading dose may be considered only for patients with severe deficiency (defined as < 20 ng/ml).

**Table 1:** Summary of core nutritional intervention components for patients with bone infections.

Nutrient category	Recommended dose/target value	Level of evidence	Evidence source	Key precautions
Energy	25–35 kcal/kg/d	Grade C	Extrapolated from ESPEN/ASPEN guidelines	Avoid overfeeding; use ideal body weight for obese
Total protein	1.5–2.0 g/kg/d	Grade C	Observational studies in bone infection + extrapolation	Reduce if eGFR < 30; monitor nitrogen balance
Glutamine	Parenteral: 0.3–0.5 g/kg/d; Enteral: 15–30 g/d	Grade B	Extrapolated from critical care RCTs	Contraindicated in severe liver failure
Arginine	Oral/enteral: 5–15 g/d	Grade C	Theoretical/indirect evidence	Use caution in uncontrolled infection
ω-3 PUFA (EPA+DHA)	Oral: 2–4 g/d	Grade C	Extrapolated from animal models and other inflammatory diseases	Use caution with anticoagulants
Vitamin D	Loading for severe deficiency (< 20 ng/ml); maintenance 800–2,000 IU/d	Grade C	Extrapolated from general repletion protocols	Monitor serum calcium and renal function
Calcium + Phosphorus	Elemental calcium: 1,000–1,200 mg/d Phosphorus: 700–1,000 mg/d	Grade D	Basic physiological data	Monitor calcium-phosphorus product in renal insufficiency
Zinc	Oral 15–25 mg/d	Grade C	Mechanistic/biomaterial evidence	Long-term >50 mg/d may cause copper deficiency
Selenium	Maintenance: 50–100 μg/d; short-term up to 200 μg/d	Grade C	Mechanistic/indirect evidence	Use caution in chronic kidney disease

Summary of core nutritional intervention protocols for patients with bone infections. Evidence levels are defined according to clinical nutrition guidelines: B, randomized controlled trial evidence; C, observational, mechanistic, or extrapolated evidence; D, insufficient or controversial evidence from basic physiological data. The B-level evidence for glutamine is derived from randomized controlled trials in the critical care nutrition field; its direct applicability to the bone infection population requires further validation. eGFR, estimated glomerular filtration rate; RCT, randomized controlled trial; PUFA, polyunsaturated fatty acid; EPA, eicosapentaenoic acid; DHA, docosahexaenoic acid; 25-(OH)D, 25-hydroxyvitamin D; CFU, colony-forming unit.

### 5.3 Calcium and phosphorus: foundation of bone mineralization

Calcium and phosphorus are the fundamental elements constituting hydroxyapatite crystals and the material basis of bone mineralization; their adequate supply is essential for bone repair following bone infections. In the bone infection state, local inflammatory responses and systemic metabolic changes can lead to calcium-phosphorus metabolic disturbances. For example, infection-induced inflammatory cytokines (such as IL-6) can upregulate RANKL expression, promoting osteoclast activation and increased bone resorption, thereby depleting local calcium and phosphorus reserves (19). Additionally, vitamin D deficiency is often accompanied by impaired calcium absorption and hypophosphatemia, further exacerbating inadequate bone mineralization, which is particularly prominent in chronic infections such as HIV, manifesting as significantly decreased bone mineral density (20). Clinical studies have also confirmed that serum albumin and calcium levels are important prognostic factors in patients with bone infections, with hypoalbuminemia significantly associated with ulcer non-healing and treatment failure (1). In orthopedic surgery, positivity for laboratory-defined nutritional risk markers (including low albumin) is closely associated with increased risks of postoperative infection, osteomyelitis, and bone grafting requirements (2). Therefore, in the treatment of bone infections, ensuring adequate calcium and phosphorus intake through nutritional support, along with correction of any coexisting vitamin D deficiency to promote their absorption, is a fundamental measure for maintaining bone mineralization, promoting bone healing, and reducing infection recurrence risk.

## 6 Immunonutrition and functional nutrients

### 6.1 Anti-inflammatory effects of omega-3 polyunsaturated fatty acids

Omega-3 polyunsaturated fatty acids (PUFAs), particularly eicosapentaenoic acid (EPA) and docosahexaenoic acid (DHA), demonstrate significant anti-inflammatory potential in inflammatory conditions such as bone infections. The core mechanism lies in the ability of EPA and DHA to competitively inhibit arachidonic acid (AA) metabolism, reducing the production of pro-inflammatory prostaglandins and leukotrienes, while generating specialized pro-resolving mediators (SPMs) such as resolvins, protectins, and maresins that actively promote inflammation resolution. These SPMs inhibit neutrophil infiltration and promote macrophage phagocytosis of apoptotic cells, accelerating the resolution of inflammation. Furthermore, omega-3 PUFAs suppress the nuclear factor-kappa B (NF-κB) signaling pathway, downregulating the expression of pro-inflammatory cytokines including tumor necrosis factor-alpha (TNF-α), interleukin-1β (IL-1β), and interleukin-6 (IL-6). In bone infection models, omega-3 PUFA supplementation has been shown to reduce inflammatory responses and protect bone tissue. For example, in a rat model of ovariectomy combined with rheumatoid arthritis, omega-3 treatment significantly reduced IL-1β, IL-6, and TNF-α levels in the temporomandibular joint while increasing the anti-inflammatory cytokine IL-10, thereby attenuating bone destruction (21). These findings indicate that omega-3 PUFAs, through their multi-target, multi-level anti-inflammatory and pro-resolving mechanisms, provide an important immunomodulatory strategy for nutritional support in patients with bone infections.

### 6.2 Probiotics and gut microbiota modulation

The balance of the gut microbiota plays a critical role in maintaining host immune homeostasis and defending against infection, and probiotics, as an effective means of modulating the gut microbiota, demonstrate considerable potential in the prevention and treatment of bone infections. The development and progression of bone infections are often accompanied by gut dysbiosis, leading to impaired intestinal barrier function and increased risk of bacterial translocation and systemic inflammation. Probiotics exert protective effects through multiple mechanisms: first, they competitively inhibit the colonization and biofilm formation of pathogens (such as Staphylococcus aureus) on intestinal and bone surfaces; second, probiotic metabolites such as short-chain fatty acids (SCFAs) and bacteriocins directly inhibit pathogen growth and enhance intestinal barrier integrity (22). More importantly, probiotics modulate host immune responses; for example, specific strains such as *Lactococcus lactis* and *Bifidobacterium bifidum* suppress NF-kB signaling and downregulate RANKL expression, thereby reducing osteoclast activation and bone resorption (23). A systematic review of gut microbiota in orthopedic surgery noted that although probiotics show some potential in modulating systemic inflammatory responses and reducing postoperative infectious complications, high-level clinical evidence for probiotics directly promoting bone healing or reducing nonunion risk remains limited, with most conclusions derived from animal experiments or small-sample observational studies (24). This evidence gap indicates that the use of probiotics as an adjunctive therapy for bone infections remains at a preliminary stage of investigation, and their adoption as a standard component of nutritional support is not yet warranted. Probiotics should instead be regarded as a promising avenue for future research, wherein modulation of the gut microbiota through probiotic supplementation may improve systemic inflammation and bone metabolism via immune regulation along the “gut–bone axis”.

### 6.3 Nucleotides and immune cell proliferation

Nucleotides, as precursors for nucleic acid synthesis, play a vital role in the metabolism and function of rapidly proliferating immune cells (such as lymphocytes, macrophages, and neutrophils). Under stress conditions such as bone infections, the body's demand for nucleotides increases dramatically, and endogenous synthesis may be insufficient to meet the rapid proliferation and differentiation needs of immune cells, making exogenous nucleotide supplementation particularly important. Nucleotides support immune function through multiple pathways: on one hand, they are essential raw materials for DNA and RNA synthesis, directly determining the capacity for immune cell clonal expansion; on the other hand, they serve as precursors for second messengers such as cyclic adenosine monophosphate (cAMP) and cyclic guanosine monophosphate (cGMP), deeply participating in immune cell activation, signal transduction, and effector function regulation. In clinical studies of bone infections, patients with positive nutritional risk markers such as hypoalbuminemia have significantly higher risks of postoperative infection and osteomyelitis (2), indirectly suggesting the potential value of comprehensive nutritional support, including nucleotides, for perioperative immune homeostasis. Although direct evidence for the role of nucleotides in bone infections is relatively limited, based on their central role in immune cell proliferation, it can be inferred that appropriate addition of nucleotides to nutritional formulas for patients with bone infections may support immune system activation and effector function, thereby enhancing the host's ability to clear pathogens and promoting infection control and tissue repair (Figure 1).

## 7 Clinical strategies for nutritional support implementation

Table 1 summarizes the recommended dosages, evidence levels, and key considerations for core nutritional intervention elements in patients with bone infections, providing clinicians with a quick reference guide before the detailed implementation pathways are systematically elaborated.

### 7.1 Nutritional risk screening and assessment tools

In the clinical management of bone infections, systematic nutritional risk screening and assessment is the first step in the implementation of effective nutritional support. At present, the commonly used screening tools include Nutritional Risk Screening 2002 (NRS-2002), subjective global assessment (SGA) and mini nutritional assessment (MNA). These tools can quickly identify patients with malnutrition or nutritional risk. The global malnutrition leadership initiative (GLIM) standard provides a standardized framework for diagnosing malnutrition. These validated tools should be used preferentially in clinical practice. It is important to distinguish among three related but distinct concepts: (1) Nutritional screening tools (e.g., NRS-2002, SGA, MNA): used to identify patients at risk of malnutrition; (2) Diagnostic criteria for malnutrition (e.g., GLIM criteria): used to confirm and grade malnutrition diagnosis (3). Inflammatory/prognostic laboratory markers (e.g., serum albumin, prealbumin, C-reactive protein): reflect both nutritional status and inflammatory burden, and are associated with clinical outcomes. In the field of bone infections, laboratory parameters have particular value due to their objectivity and quantifiability. Studies have shown that positivity for laboratory-defined nutritional risk markers (serum albumin ≤ 3.5 g/dl and/or lymphocyte count ≤ 1.5 × 10^3^/μl) is associated with significantly increased risks of acute respiratory failure, venous thromboembolism, transfusion requirements, postoperative infection, wound dehiscence, sepsis, and acute kidney injury within 90 days after humeral shaft fracture surgery, with these risks persisting through 2 years of follow-up, including infection, implant removal, osteomyelitis, and bone grafting (2). Furthermore, in patients with pressure ulcer-related pelvic osteomyelitis, low albumin (< 25 g/L) and low hemoglobin (< 111 g/L) were identified as independent risk factors for ulcer non-healing (1). Although these data are derived from an orthopedic trauma population rather than a bone infection cohort, they suggest that similar laboratory markers may be useful for risk stratification in patients with bone infections. Therefore, we recommend incorporating objective parameters including serum albumin, prealbumin, transferrin, and lymphocyte count into a routine screening system, in combination with subjective assessment tools, to achieve precise stratification of nutritional status in patients with bone infections, while recognizing that direct validation of these markers in this population is still lacking. When interpreting associations between nutritional markers and clinical outcomes in observational studies, it is important to recognize potential confounding factors. Hypoalbuminemia and other nutritional risk markers may be influenced by age, diabetes, renal disease, systemic inflammation, malignancy, obesity, frailty, smoking, vascular insufficiency, and infection severity. Therefore, observed associations should not be automatically interpreted as causal evidence that nutritional intervention will improve prognosis. Rather, they should be viewed as risk stratification signals that identify patients who may benefit from more comprehensive nutritional assessment.

### 7.2 Selection of enteral vs. parenteral nutrition

In selecting the route of nutritional support for patients with bone infections, enteral nutrition (EN) is generally recommended as the preferred option due to its physiological compatibility, ability to maintain intestinal barrier function, reduction of bacterial translocation, and lower infectious complication rates. However, patients with bone infections often have impaired gastrointestinal function due to trauma, surgery, or systemic inflammatory responses, in which case parenteral nutrition (PN) may serve as a supplement or alternative. Clinical decisions should be based on the patient's specific condition, including gastrointestinal function status, infection severity, and surgical type. For patients who can tolerate enteral feeding, EN should be initiated as early as possible and gradually increased to target doses; if EN cannot meet energy and protein requirements (typically defined as EN providing less than 60% of target for more than 7 days), PN should be combined. Notably, in bone infection treatment, the goal of nutritional support is not only to correct malnutrition but also to provide substrate support for immune function and bone healing. Studies have shown that in patients with pressure ulcer-related osteomyelitis, nutritional optimization (including efforts to improve nutritional status in patients with hypoalbuminemia, while recognizing that albumin levels may also reflect inflammatory status and are not a direct measure of protein intake alone) is one of the key measures for improving prognosis (1). Furthermore, for patients with severe infection or sepsis, the timing and composition of PN require more careful consideration to avoid overfeeding and metabolic complications. Therefore, a multidisciplinary team approach (including dietitians, infectious disease specialists, and orthopedic surgeons) should be established in clinical practice to dynamically adjust the nutritional route based on the patient's daily metabolic status and tolerance.

### 7.3 Perioperative nutritional support protocol

The perioperative period is a critical window for nutritional intervention in patients with bone infections, and appropriate nutritional support protocols can significantly reduce surgical complications and promote postoperative recovery. Preoperative nutritional assessment should be performed as early as possible. Retrospective evidence indicates that patients with preoperative nutritional risk markers (such as albumin ≤ 3.5 g/dl) have significantly higher risks of postoperative infection and osteomyelitis (2). Based on this, preoperative nutritional optimization for screen-positive patients is a reasonable clinical strategy. Regarding the specific timing of prehabilitation, there is currently no high-level evidence in the bone infection field supporting a uniform “7–10 day” window. Referencing general recommendations from enhanced recovery after surgery (ERAS) and critical care nutrition guidelines, preoperative intensified nutritional support may be initiated 5–7 days prior to surgery, but the applicability of this timeframe in patients with bone infections requires prospective studies for confirmation. Clinical practice should be dynamically adjusted based on patient tolerance and metabolic status. Intraoperatively and postoperatively, ERAS principles should be followed: clear fluids via enteral nutrition can be attempted 6 h after recovery from anesthesia, with gradual escalation to target volumes within 24–48 h. For patients with bone infections, according to the inference of general surgery and intensive care nutrition guidelines (evidence level C), postoperative nutritional support can reasonably set the protein supply at 1.5–2.0 g / kg / d and the energy supply at 25–35 kcal / kg / d. However, it has not been specifically verified in the bone infection population through randomized controlled trials. These targets should be adjusted based on the patient's clinical response, renal function, and tolerance, and should not be applied rigidly without ongoing reassessment. Studies have confirmed that in patients after humeral shaft fracture surgery, those with positive laboratory nutritional risk markers have significantly higher rates of wound complications and osteomyelitis, suggesting that perioperative nutritional intervention is critical for preventing infection-related complications (2). Additionally, for patients with pressure ulcer-related pelvic osteomyelitis, postoperative soft tissue coverage attempts combined with nutritional optimization can improve ulcer healing rates (1). Therefore, perioperative nutritional support protocols should be individualized, including preoperative nutritional optimization, early postoperative EN initiation, and correction measures for specific complications (such as hypoalbuminemia), with continuous monitoring of nutritional parameters and clinical outcomes.

### 7.4 Interaction between nutritional support and antibiotic therapy

Nutritional support and antibiotic therapy have synergistic effects in bone infection management, with the core mechanism being that nutritional status directly affects host immune function and antibiotic pharmacokinetics. Malnutrition, particularly protein-energy malnutrition, can impair the function of immune cells (such as neutrophils, macrophages, and T lymphocytes), thereby reducing the body's ability to clear pathogens and increasing the risk of antibiotic treatment failure. Studies have shown that in patients with pressure ulcer-related pelvic osteomyelitis, hypoalbuminemia is significantly associated with ulcer non-healing and treatment failure, and nutritional optimization (including correction of hypoalbuminemia) is an important measure for improving prognosis (1). Furthermore, specific nutrients such as glutamine, arginine, and omega-3 polyunsaturated fatty acids have immunomodulatory effects that may enhance the bactericidal efficacy of antibiotics. In animal models, probiotics (such as Enterococcus faecium and Bacillus species) have been shown to significantly reduce the incidence of lameness due to bacterial chondronecrosis with osteomyelitis through improved gut health and reduced bacterial translocation, suggesting that gut microbiota modulation may enhance anti-infection efficacy through the immune-nutrition axis (25). On the other hand, antibiotic treatment itself may affect nutritional metabolism; for example, prolonged broad-spectrum antibiotic use can lead to gut dysbiosis, thereby affecting nutrient absorption. Therefore, clinical practice should integrate nutritional support with antibiotic treatment strategies, including: ensuring adequate protein and energy intake to maintain immune function during antibiotic therapy; monitoring and correcting micronutrient deficiencies (such as zinc and vitamin D); and considering the use of probiotics or synbiotics to mitigate antibiotic-associated gut dysbiosis. This integrated strategy holds promise for improving the success rate of bone infection treatment. This integrated strategy holds promise for improving the success rate of bone infection treatment, although whether nutritional optimization can reduce the emergence of antibiotic resistance remains speculative and requires dedicated investigation.

## 8 Current challenges and future directions

Although the theoretical value of nutritional support in bone infection treatment has gained broad recognition, several critical challenges must be acknowledged.

First, there is a severe paucity of high-level clinical evidence. Most current recommendations derive from observational studies or extrapolation from critical care nutrition guidelines; large-scale randomized controlled trials (RCTs) specifically targeting osteomyelitis and PJI patients are lacking, making it difficult to support the development of standardized guidelines.

Second, the double-edged sword effect of nutritional interventions. Immunonutrients such as arginine, while enhancing host bactericidal capacity, may theoretically provide proliferative substrates for intracellular pathogens through metabolic shunting (such as the arginase pathway in certain pathogens)—a risk that has not been adequately evaluated in Staphylococcus aureus infections. Overaggressive energy supply may also induce hyperglycemia and refeeding syndrome, paradoxically worsening the immune microenvironment.

Third, the biological basis for individualized nutritional protocols remains unclear. The metabolic profile of patients with bone infections is influenced by multiple factors including pathogen species, antibiotic duration, comorbidities, and genetic background; current universal “one-size-fits-all” recommended dosages (such as 1.5–2.0 g/kg/d protein) lack biomarkers for dynamic monitoring and real-time adjustment.

Future directions should focus on: (1) conducting multicenter RCTs to establish the causal effects of nutritional interventions; (2) utilizing metabolomics and single-cell sequencing technologies to map the “nutrition-immunity-bone repair” spatiotemporal atlas of the bone infection microenvironment; and (3) exploring precision microbiome intervention strategies based on the “gut-bone axis.”

## 9 Conclusion

The treatment of bone infections has evolved from a singular focus on surgical debridement and antibiotic application toward a comprehensive multidisciplinary management model, in which nutritional support plays an important supportive role. From the review presented herein, it is evident that the hypermetabolic, hypercatabolic, and micronutrient-depleting characteristics of the bone infection state directly lead to delayed bone healing, immunosuppression, and uncontrolled inflammation, providing clear pathophysiological rationale for nutritional intervention. Proteins, specific amino acids, vitamin D, and zinc not only participate in bone matrix synthesis as structural substrates but also establish dynamic equilibrium between infection control and tissue repair through modulation of immune cell activity and inflammatory signaling pathways.

In clinical practice, there is some divergence in perspectives regarding the timing and dosage of nutritional support: some emphasize that early aggressive nutritional intervention can significantly reduce complications, while others caution that overfeeding may exacerbate metabolic burden. The balance point of this divergence lies in individualized assessment based on standardized nutritional risk screening tools (such as NRS-2002) rather than blind application of uniform protocols. Meanwhile, enteral nutrition as the preferred route has achieved broad consensus, but in patients with gastrointestinal insufficiency or hypermetabolic demands, appropriate supplementation with parenteral nutrition remains indispensable.

In the future, with the advancement of metabolomics technologies, precision nutrition protocols are expected to break through the current “one-size-fits-all” limitations, enabling customized adjustment of amino acid ratios, vitamin dosages, and immunonutrient (such as glutamine and arginine) supplementation through dynamic monitoring of the patient's metabolic profile. This direction will propel bone infection treatment from empirical support toward evidence-based, individualized practice, potentially contributing to improved clinical outcomes, including reduced disease duration, lower recurrence rates, and enhanced quality of life, although these benefits remain to be confirmed by prospective studies.

Importantly, the majority of recommendations presented in this review are derived from observational data, mechanistic studies, or extrapolation from other patient populations. High-quality randomized controlled trials specifically targeting nutritional interventions in osteomyelitis and PJI are urgently needed to confirm these proposed benefits. The recommendations provided herein should therefore be interpreted as practice frameworks requiring individualization based on patient-specific factors, rather than as fixed protocols.
